# High mobility group box 1 (HMGB1) and anti-HMGB1 antibodies and their relation to disease characteristics in systemic lupus erythematosus

**DOI:** 10.1186/ar3332

**Published:** 2011-05-06

**Authors:** Deena A Abdulahad, Johanna Westra, Johannes Bijzet, Pieter C Limburg, Cees GM Kallenberg, Marc Bijl

**Affiliations:** 1 Department of Rheumatology and Clinical Immunology, University Medical Center Groningen, University of Groningen, PO Box 30.001, 9700 RB Groningen, The Netherlands

## Abstract

**Introduction:**

High Mobility Group Box 1 (HMGB1) is a nuclear non-histone protein. HMGB1, which is secreted by inflammatory cells and passively released from apoptotic and necrotic cells, may act as a pro-inflammatory mediator. As apoptotic cells accumulate in systemic lupus erythematosus (SLE), HMGB1 levels might be increased in SLE. HMGB1 may also serve as an autoantigen, leading to the production of anti-HMGB1 antibodies. In this study we determined levels of HMGB1 and anti-HMGB1 in SLE patients in comparison to healthy controls (HC) and analysed their relation with disease activity.

**Methods:**

The study population consisted of 70 SLE patients and 35 age- and sex-matched HC. Thirty-three SLE patients had quiescent disease, the other 37 patients were selected for having active disease. Nineteen of these had lupus nephritis. HMGB1 levels were measured with both Western blot and ELISA. Anti-HMGB1 levels were measured by ELISA. Clinical and serological parameters were assessed according to routine procedures.

**Results:**

HMGB1 levels in SLE patients could be measured reliably by Western blotting only, and were significantly increased compared to HC. During active disease HMGB1 levels increased, in particular in patients with renal involvement. Serum HMGB1 levels correlated with SLEDAI, proteinuria, and anti-dsDNA levels, and showed a negative correlation with complement C3. Anti-HMGB1 levels were significantly increased in SLE patients compared to HC, and positively correlated with HMGB1 levels.

**Conclusions:**

Levels of HMGB1 in the sera of SLE patients, in particular in those with active renal disease, are increased. Serum HMGB1 levels are related to SLEDAI scores and proteinuria, as well as to levels of anti-HMGB1 antibodies. These findings suggest that besides HMGB1, HMGB1-anti-HMGB1 immune complexes play a role in the pathogenesis of SLE, in particular in patients with renal involvement.

## Introduction

Systemic Lupus Erythematosus (SLE) is a systemic autoimmune disease characterised by involvement of multiple organ systems. Its aetiology is largely unknown; however, genetic and environmental factors are proposed to contribute to breaking tolerance, resulting in the production of a variety of antibodies directed to self-components [1]. These autoantibodies can form immune complexes which can be deposited in many tissues like skin and kidney [2-5]. Antinuclear autoantibodies (ANA) and especially autoantibodies against dsDNA (double stranded DNA) represent a serological hallmark of SLE, and may serve as indicators for disease activity and severity [6,7].

Pathophysiological mechanisms involved in breaking tolerance against self components are not fully understood. However, in the past few years disturbance in the clearance of apoptotic cells has been reported, and it has been suggested that apoptotic cells can serve as a source of autoantigens [8-10].

High mobility group box 1 (HMGB1), originally recognised as a DNA binding protein, has recently been identified as a damage associated molecular pattern (DAMP) [11,12]. Inside the cell, it binds to DNA and participates in many nuclear functions but once released it is involved in inflammatory functions [13,14]. HMGB1 is actively released from LPS-, TNF α- and IL-1 activated monocytes and macrophages and from other cell types [13,15-17]. In addition, HMGB1 is released from damaged dying cells during necrosis as well as during the late phase of apoptosis [18,19]. Extracellular HMGB1 exerts its biological actions through binding to cell-surface receptors, such as RAGE (receptor of advanced glycation end products), TLR2, TLR4, and the intracellular receptor TLR9 [20-23].

Recent studies have shown an association between HMGB1 and chronic inflammation and autoimmunity. High levels of HMGB1 have been found in several rheumatic diseases such as RA and Sjogren's syndrome [24-26]. Little is known about the involvement of HMGB1 in the pathogenesis of SLE. In SLE, HMGB1 was demonstrated to be associated with nucleosomes released from apoptotic cells and to contribute to the immunostimulatory effect of nucleosomes [27]. In addition, HMGB1 has been found to be significantly elevated in lupus sera and has been regarded as one of the components in DNA-containing immune complexes that enhance cytokine production through TLR9 or RAGE ligation [23,28,29]. Interestingly, in addition to anti-dsDNA antibodies (anti-double stranded DNA antibodies), antibodies against HMGB1 have been detected in sera from SLE patients. As a result, HMGB1 has been identified as new auto-antigen in SLE [28]. The relation between levels of HMGB1, levels of antibodies to HMGB1, disease activity and disease manifestations of SLE has not been evaluated extensively.

In this study we determined serum levels of HMGB1 and anti-HMGB1 antibodies in a large group of SLE patients in relation to disease activity and disease characteristics, with focus on renal involvement.

## Materials and methods

### Patients

The study population consisted of 70 SLE patients and 35 age- and sex-matched healthy controls (HC) following the ethical consent approved by the human ethics committee. All patients provided the informed consent and fulfilled the criteria of the American College of Rheumatology for SLE. Fifty-eight female (83%) and 12 (17%) male patients were included; ages ranged from 18 to 73 years (mean 41.1 ± SD 13.5 yrs). Of the 70 SLE patients 33 were patients with quiescent disease visiting the outpatient clinic. The other 37 patients were selected for the presence of active disease. Clinical data were obtained from all patients and the study was approved by the human ethics committee. Disease activity at the time of blood sampling was assessed using SLE Disease Activity Index (SLEDAI). Patients with SLEDAI ≥4 were considered active, and patients with a SLEDAI score <4 were considered to have quiescent disease. Nineteen of the 37 patients with active disease had renal involvement and the remaining 18 patients had extra-renal disease activity only. Levels of anti-dsDNA, C-reactive protein (CRP), creatinine (Cr), glomerular filtration rate (GFR), and complement factors (C3, C4) were determined by routine techniques. The control group comprised 35 healthy volunteers, 27 women (77%) and 8 men (23%), aged 21 to 64 (mean 38.4 ± SD 11.9 yrs). Data are summarized in Tables 1 and 2.

**Table 1:** State of SLE patients included in the study

	*SLE patients*	
	**Quiescent disease (*n *= 33)**	**Active disease (*n *= 37)**
No. male/female	5/28	7/30
Age (years), median (range)	45 (19 to 73)	37 (18 to 73)
SLEDAI, median (range)	2 (0 to 4)	10 (5 to 16) ***
Anti-dsDNA (E/ml), median (range)	25 (3 to 408)	230 (3 to 6,683) ***
C3(g/l), median (range)	0.99 (0.54 to 1.46)	0.73 (0.21 to 1.68) ***
C4 (g/l), median (range)	0.15 (0.06 to 0.28)	0.12 (0.02 to 0.30) *
CRP (g/I), median (range)	5 (5 to 27)	6 (1 to 92)
No. with/without treatment	31/2	29/8
Users of Prednisone (%) Dose (mg/day), median (range)	19 (58%) 10 (2.5 to 50)	21 (57%) 7.50 (2.5 to 100)
Users of Azathioprine (%) Dose (mg/day), median (range)	8 (24%) 150 (50 to 150)	5 (14%) 100 (75 to 150)
Users of Hydroxychloroquine (%) Dose (mg/day), median (range)	19 (58%) 400 (150 to 600)	16 (43%) 400 (200 to 1,200)

* Difference between quiescent and active SLE patients (* *P < 0.05*, ** *P < 0.001*, ***P < 0.0001).

**Table 2:** Characteristics of active SLE patients with renal and non-renal involvement

	*Renal active (n = 19)*	*Non-renal active (n = 18)*
No. male/female	5/14	2/16
Age (yrs), median (range)	38 (18 to 73)	37 (19.60)
SLEDAI, median (range)	12 (6 to 16) ***	8 (5 to 10)
Anti-dsDNA (E/ml), median (range)	230 (7 to 6,683)	234 (3 to 1,000)
C3 (g/l), median (range)	0.58 (0.21 to 1.12) *	0.91 (0.37 to 1.68)
C4 (g/l), median (range)	0.10 (0.04 to 0.30)	0.13 (0.02 to 0.27)
CRP (g/l), median (range)	5 (1 to 81)	9.5 (3 to 92)
No. with/without treatment	17/2	12/6
Users of Prednisone (%) Dose (mg/day), median (range)	14 (78%) 6.25 (2.5 to 100)	7 (39%) 7.50 (5 to 20)
Users of Azathioprine (%) Dose (mg/day), median (range)	2 (11%) 87.5 (75 to 100)	3 (17%) 125 (100 to 150)
Users of Hydroxychloroquine (%) Dose (mg/day), median (range)	8 (42%) 400 (200 to 400)	8 (44%) 400 (200 to 1,200)

* Difference between renal active patients and non-renal active SLE patients. (*P < 0.05, *** *P < 0.0001).*

### ELISA for serum HMGB1

HMGB1 levels in the sera of patients and HC were quantified using a commercial enzyme-linked immunosorbent assay (ELISA) kit, according to manufacturer's instructions (Shino-test, Sagamihara, Kanagawa, Japan).

### Western blot for serum HMGB1

Sera (3 μl) from SLE patients and HC were diluted in 72 μl SDS buffer (0.063 M Tris.HCl pH 6.8, 2% SDS, 10% glycerol, 0.015% BromePhenol Blue, and 5% ß-mercaptoethanol) and heated at 98°C for five minutes. Next, proteins were resolved on 12.5% SDS-PAGE gel (Criterion gel BioRad, Veenendaal, The Netherlands) and transferred to polyvinylidene fluoride membrane (Millipore, Amsterdam, The Netherlands) followed by blocking with Odyssey buffer (LI-COR Biotechnology, Lincoln, NE, USA) at room temperature for one hour. Membranes were then incubated with anti-HMGB1 mouse monoclonal antibody (1:250; R&D Systems, Abingdon, UK) overnight in Odyssey buffer diluted with PBS at 4°C. After washing with Tris buffered saline with Tween-20 (TBST), membranes were incubated with polyclonal goat anti-mouse IgG labelled with IRDye800 (1: 5000; LI-COR Biotechnology) for one hour. Blots were scanned with Odyssey infrared Imaging System (LI-COR Biotechnology) and then analysed with the Odyssey software (version 2.1). A standard sample was prepared by adding SDS buffer to human keratinocyte HaCaT cells and was included in each blot as an internal control. In each blot, levels of HMGB1 were expressed as values of fluorescence intensity and were calculated against the standard sample.

### Serum IgG depletion

Serum IgG was depleted using HiTrap Protein G HP column, according to the manufacturer's instructions (GE Healthcare Europe, Diegem, Belgium).

### ELISA for anti-HMGB1 antibodies

Autoantibodies against HMGB1 were measured by an in-house developed ELISA. Briefly, Maxisorp polystyrene 96-wells plates were coated with 50 μL per well of rHMGB1 (R&D Systems, Abingdon, UK) at 1 μg/ml in PBS and incubated overnight at 4°C. Plates were blocked with 5% BSA in PBS for two hours. Serum samples, diluted 1:50 in incubation buffer, were added in duplicate (100 μl/well) and incubated for two hours at room temperature. After five washes, 100 μl HRP-conjugated goat anti-human IgG (SouthernBiotech, Birmingham, AL, USA), diluted 1:3,000, was added to each well and incubated for one hour at room temperature. After washing, bound antibodies were detected using 3,3',5,5'-tetramethylbenzidine dihydrochloride (TMB). The reaction was stopped with 2 M sulphuric acid and the absorbance was measured at 450 nm using a microplate-spectrophotometer (Vmax, Molecular Devices, Sunnyvale, CA, USA). Anti-HMGB1 antibody levels were expressed in Optical Density values.

### Statistical analysis

Data are presented as median (range) unless stated otherwise. Statistical analysis was performed by using the statistical package Graph Pad Prism, version 3.02 (Graph Pad Software Inc., San Diego, CA, USA). A Student *t *test or a Mann-Whitney test was used for comparison of different groups as appropriate. Spearman rank correlation was used to assess correlations. A *P-*value < 0.05 was considered significant.

## Results

### Serum HMGB1 levels by ELISA

**Figure 1:**
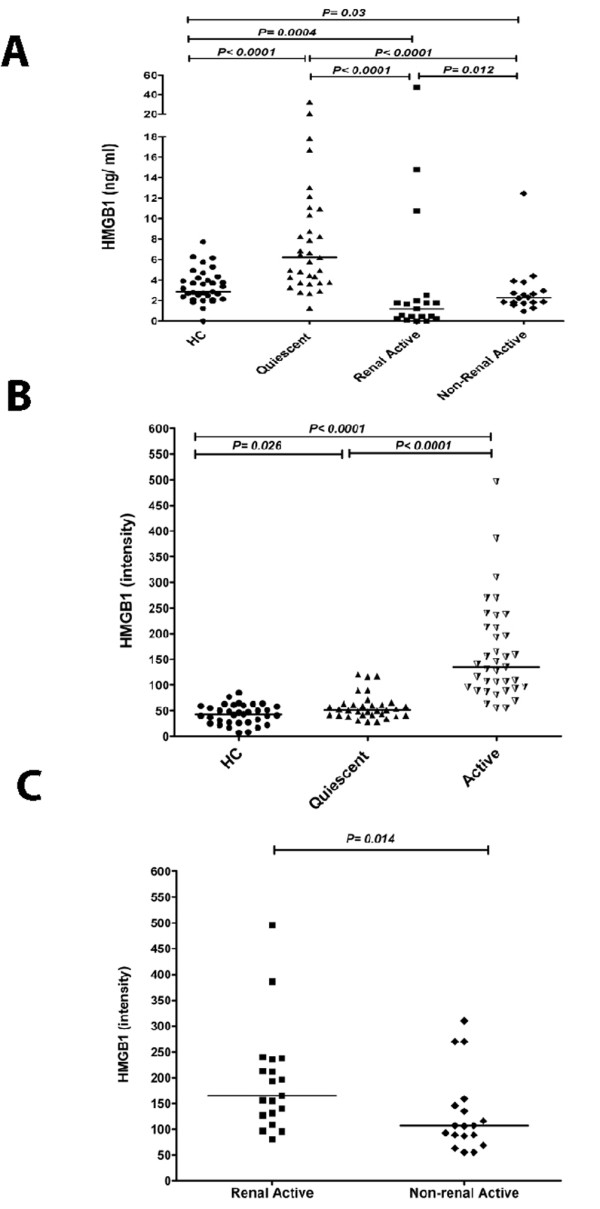
**HMGB1 concentrations in sera from SLE patients and healthy controls (HC)**. **A**) Serum HMGB1 levels in SLE patients and HC using ELISA. Horizontal lines represent the median. **B**) Serum HMGB1 levels of SLE patients and HC measured by Western blot. **C**) HMGB1 levels measured by Western blot in active patients with renal manifestations and with those without renal manifestations.

HMGB1 levels in serum samples from patients and HC were assessed using a commercial ELISA kit. With this kit we found increased HMGB1 levels in quiescent SLE patients (6.2 ng/ml (1.3 to 32.3)) compared to HC (2.9 ng/ml (0 to 7.7)). However, in selected patients with active disease, both those with renal and non-renal disease activity had decreased HMGB1 levels (1.2 ng/ml (0 to 47.2)) and 2.3 ng/ml (0.95 to 12.5), respectively) compared to HC. Levels of HMGB1 in patients with active disease were also significantly lower in comparison to quiescent patients. In addition, HMGB1 levels were significantly decreased in patients with renal involvement compared to those without (Figure 1A).

Considering the possibility that serum proteins and antibodies interfere with HMGB1 in the ELISA system, we measured serum HMGB1 levels as well by Western blot.

### Serum HMGB1 levels measured by Western blot

HMGB1 levels in serum samples from SLE patients and HC were additionally assessed using a Western blot assay. In quiescent SLE patients, HMGB1 levels were significantly increased (51 (28 to 121)) compared to HC (43 (7 to 85)), while in patients who had active disease HMGB1 levels were even higher (135 (55 to 496)), (Figure 1B). Within the group of active patients those with renal manifestations had higher HMGB1 levels (165 (81 to 496)) compared to active patients with non-renal manifestations (107 (55 to 310)) (Figure 1C).

**Figure 2:**
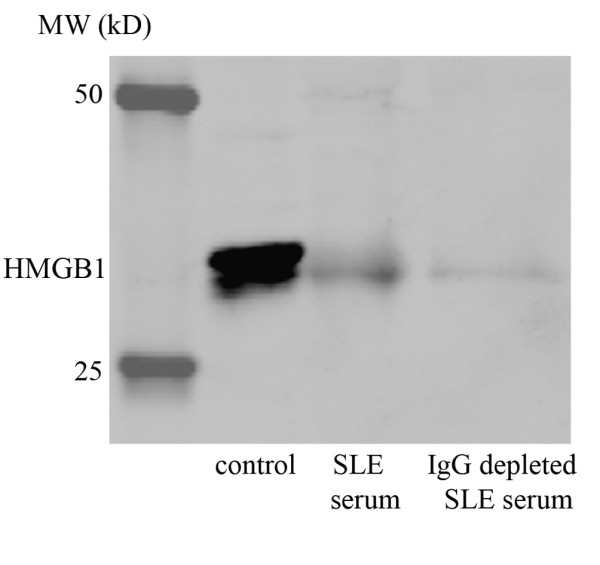
**Serum HMGB1 after IgG depletion**. HMGB1 levels are decreased in serum depleted of IgG from an SLE patient. The first lane represents the standard control consisting of human keratinocyte HaCaT cells; the second lane represents the HMGB1 amount in the serum of a SLE patient before IgG depletion and in the third lane the HMGB1 amount after IgG depletion in the serum of same patient is shown.

HMGB1 results obtained by Western blot were further used for correlations and conclusion as we interpreted the discordant results obtained by ELISA as a possible effect of immune complex formation. To confirm the interference of immune complexes with the detection of serum HMGB1 by ELISA, we depleted IgG from serum of patients using a protein G column. HMGB1 levels decreased when serum was depleted of IgG indicating that serum HMGB1, at least in part, is present in immune complexes which are not detected using ELISA (Figure 2). However, due to denaturation and high temperatures in Western blotting, immune complexes are dissociated and the HMGB1 band exists of free and previously complexed HMGB1.

### Antibodies against HMGB1

**Figure 3:**
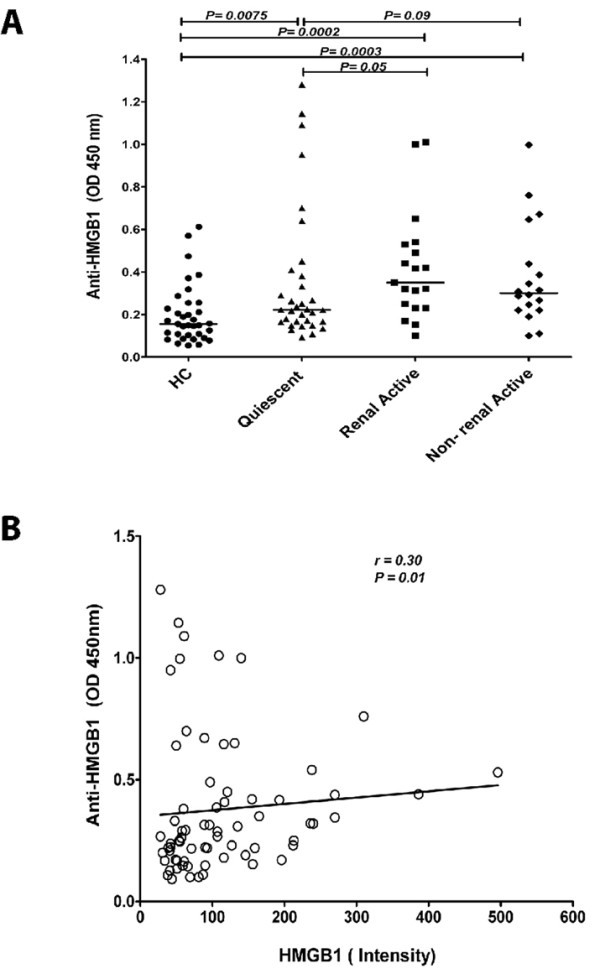
**Anti-HMGB1 levels in SLE patients and their relation to HMGB1**. **A**) Anti-HMGB1 levels in SLE patients compared to HC. Horizontal lines represent the levels expressed as median. **B**) Positive correlation between levels of HMGB1 and anti-HMGB1 antibodies.

In-house developed ELISA levels of antibodies against HMGB1 were measured in healthy controls and in SLE patients. Anti-HMGB1 levels were significantly increased in quiescent SLE patients (0.22, (0.09 to 1.30)) compared to HC (0.16 (0.05 to 0.61). In patients with renal (0.35, (0.10 to 1.01)) and non-renal 0.30, (0.10 to 0.99)) manifestations during active periods of the disease, anti-HMGB1 levels were significantly increased in comparison to HC. Anti-HMGB1 levels were significantly higher in active patients compared to quiescent patients (Figure 3A). In addition, there was a positive correlation, albeit weak, between levels of HMGB1 and anti-HMGB1 antibodies in the total patient group (*P *= 0.018, r = 0.28) (Figure 3B).

### Correlations of HMGB1 and anti-HMGB1 antibody levels with clinical and serological findings

**Figure 4:**
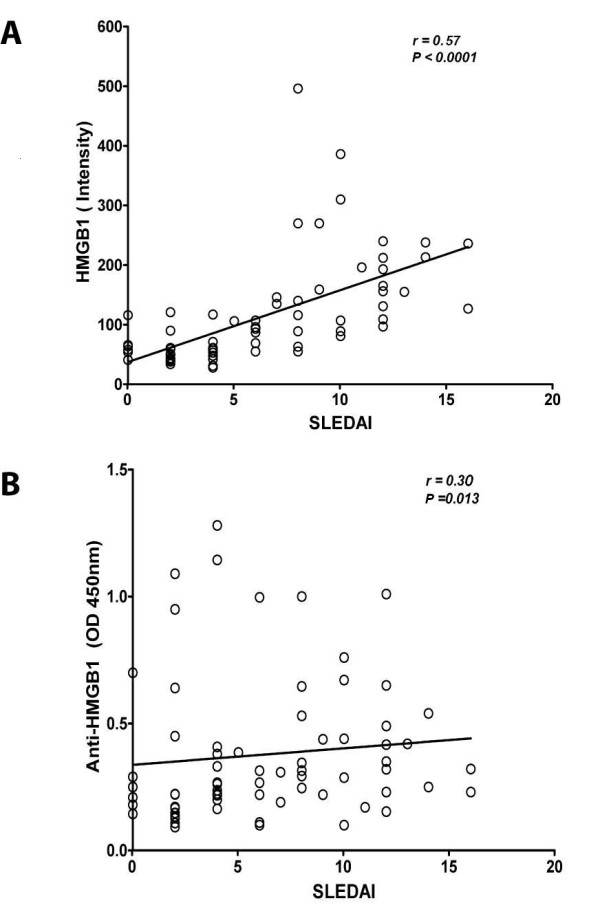
**Correlation of SLEDAI scores with levels of HMGB1 and anti-HMGB1 antibodies in SLE patients**. **A**) SLEDAI scores correlate positively with HMGB1 levels as well as with **B**) Anti-HMGB1 antibody levels.

**Figure 5:**
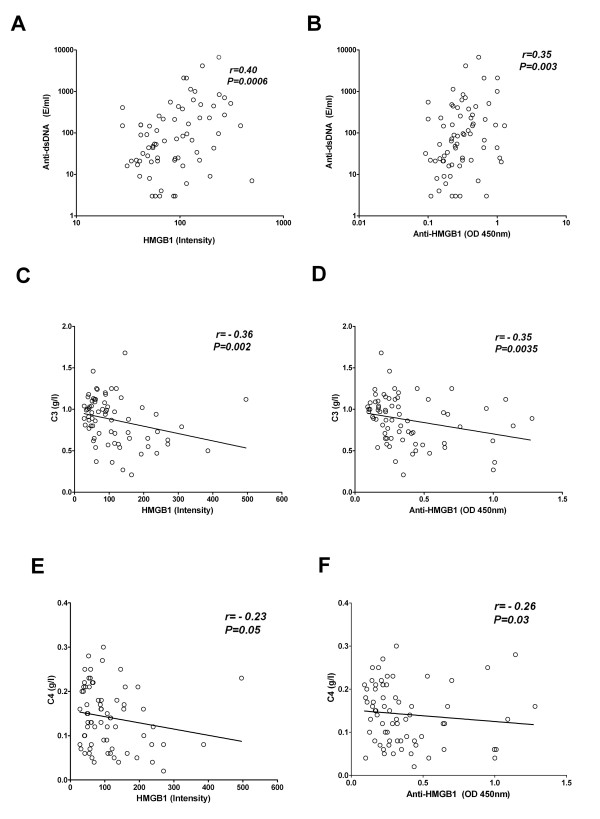
Correlations of HMGB1 and anti-HMGB1 antibodies with anti-dsDNA antibodies, C3 and C4 in SLE patients

As HMGB1 might be a marker of certain disease activity in SLE we evaluated whether levels of HMGB1 and antibodies against HMGB1 were associated with clinical and serological parameters in SLE patients. Analysing data of the total group of patients we observed a correlation between HMGB1 levels and SLEDAI (*P *< 0.0001, r = 0.57) (Figure 4A). Also, anti-HMGB1 levels showed a significant correlation with SLEDAI (*P *= 0.013, r = 0.30) (Figure 4B). HMGB1 levels correlated with anti-dsDNA (*P *= 0.0006, r = 0.40). Similarly, anti-HMGB1 antibodies showed a correlation with anti-dsDNA levels (*P *= 0.003, r = 0.35) (Figure 5A, B). Complement proteins are involved in the pathogenesis of SLE and are considered biomarkers for disease activity. Therefore, we investigated the correlation of these factors with HMGB1 and anti-HMGB1 levels. We observed a negative correlation in the total SLE group between C3, C4 and HMGB1 *(P *= 0.002, r = -0.36 and *P *< 0.05, r = -0.23 respectively) (Figure 5C, E). Also, anti-HMGB1 antibodies showed a significant negative correlation with C3 and C4 (*P *= 0.0035, r = -0.3*5 *and *P *= 0.03, r = -0.26 respectively) (Figure 5D, F).

**Figure 6:**
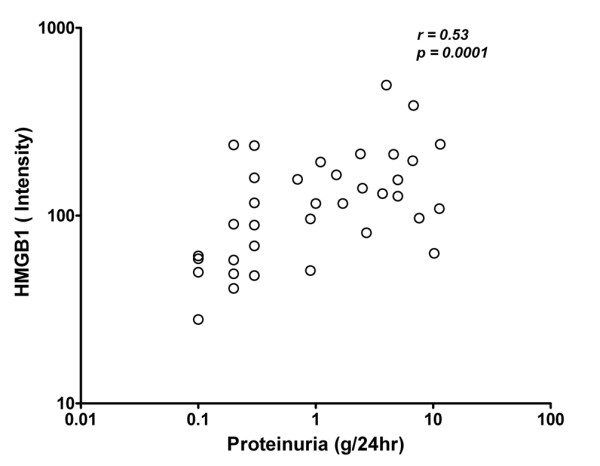
Correlation of HMGB1 levels with proteinuria in SLE patients

Finally, we assessed whether HMGB1 was related to kidney involvement. No correlation was observed between levels of HMGB1 and creatinine, nor with estimated Glomerular Filtration Rate (eGFR) (data not shown). However, in the 33 patients with proteinuria, a correlation was found between HMGB1 levels and the amount of proteinuria (*P *= 0.0001, r = 0.53) (Figure 6).

## Discussion

In this study we showed that both HMGB1 levels and anti-HMGB1 levels are increased in SLE patients, and are related to SLE disease activity scores and serological measures of disease activity [24-26,29]. Recent studies showed increased levels of serum HMGB1 and anti-HMGB1 in several autoimmune diseases including SLE. However, the relation between levels of HMGB1 and anti-HMGB1 antibodies has not been evaluated in a large group of SLE patients. We measured serum HMGB1 using two methods, ELISA and Western blot. The ELISA kit used in this study has been used to detect serum HMGB1 levels mainly in other chronic inflammatory diseases [30,31]. Urbonaviciute *et al*. showed that there was a discrepancy in serum/plasma HMGB1 results obtained from their in-house developed ELISA and Western blot in SLE patients due to possible interference of HMGB1-binding antibodies and unidentified serum proteins with HMGB1 [32]. Indeed, we could show that IgG depletion decreased the amount of HMGB1 in sera from lupus patients, suggesting that at least part of HMGB1 is complexed with anti-HMGB1 antibodies. In this study prevalence of serum HMGB1 in patients obtained by ELISA were low in accordance with Ma *et al.*, who also detected serum HMGB1 levels by the same kit [33]. To avoid possible interference of immune-complex formation or other serum proteins in ELISA, we assessed HMGB1 levels in all SLE and HC using Western blot. HMGB1 levels were significantly increased in all SLE patients compared to HC and HMGB1 levels were highest in patients with active disease.

Our Western blot results are in line with Li *et al.*, who also found elevated levels of serum HMGB1 in Chinese SLE patients [29]. Increased expression of HMGB1 has also been reported in other autoimmune diseases particularly in RA. It has been shown that HMGB1 is increased in synovial tissues where inflammation exists; however, HMGB1 levels in sera of RA patients were not detectable [32]. It is speculated that in RA activated inflammatory cells such as macrophages in the synovial tissue are the source of HMGB1 [34-36]. In SLE, the increased HMGB1 levels found in serum might also be the product of activated inflammatory cells but might be a product of uncleared apoptotic cells as well. Overall, our results and previous findings indicate that HMGB1, as a pro-inflammatory cytokine, could be an important player in the inflammatory processes in SLE and might be specific for the disease.

In accordance with Li *et al.*, HMGB1 levels were correlated positively with SLEDAI. The study by Li *et al.*, did not demonstrate an association of HMGB1 with specific organ involvement [29]. HMGB1 has been implicated in the pathogenesis of kidney diseases in patients with lupus nephritis and antineitrophil cytoplasmic antibody-associated vasculitis with renal involvement, and also has been shown to promote granulomatous nephritis in an adenine-fed rat model [31,37-41]. In this model it was shown that granuloma formation was associated with high HMGB1 expression and up-regulation of HMGB1 receptors such as RAGE and TLR4. Therefore, we investigated if levels of HMGB1 are related to kidney involvement in SLE. Serum HMGB1 levels were higher in patients with renal involvement compared to patients without renal involvement. In chronic kidney disease, HMGB1 has been shown to correlate with renal function [37]. This could not be demonstrated in our SLE patients as no correlation could be demonstrated between the presence of HMGB1 and renal function parameters, such as serum creatinine, and eGRF. However, we could observe a positive correlation between serum HMGB1 levels and proteinuria. Such association has been shown in a murine adenovirus accelerated SLE model [42]. Interestingly, antagonizing HMGB1 by administration of anti-HMGB1 mAb IA-4 in this model markedly inhibited proteinuria and led to kidney protection. Combined, all these data indicate that HMGB1 might be an important molecule involved in renal pathology.

Besides the role of HMGB1 as a pro-inflammatory protein, it has been reported that antibodies against HMGB1 occur in SLE patients. In line with published findings of Hayashi *et al.*, anti-HMGB1 antibodies were significantly elevated in all SLE patients compared to healthy controls [28]. Moreover, our results indicate, similarly to HMGB1, that anti-HMGB1 antibodies correlate positively with SLEDAI in SLE patients suggesting a pathological role of these antibodies. Until now, no reports are available on the relation between HMGB1 and anti-HMGB1 antibodies. Our results showed a positive correlation between HMGB1 and anti-HMGB1 antibodies in SLE patients. The pathogenic or neutralizing role of these antibodies is still debated. The interaction between anti-HMGB1 antibodies and HMGB1 in the pathogenesis of SLE needs further study. In addition, we found that levels of anti-HMGB1 antibodies and anti-dsDNA antibodies are correlated suggesting that antibodies against HMGB1 and nucleosomes might be produced as a result of, possibly, concurrent with B cell activation by HMGB1 and nucleosomes.

In summary, this study shows that serum HMGB1 is significantly increased in SLE patients, in particular those with renal involvement, and correlates with proteinuria, concurrent with anti-HMGB1 antibodies were detected. HMGB1 and anti-HMGB1 antibodies may serve as a new biomarker to be used in the diagnosis and assessment of disease activity in SLE patients, as well to predict the disease flares. However, the differential role of HMGB1, anti-HMGB1 and their complexes in the pathogenesis of SLE, in particular in patients with renal involvement needs further investigation. Therapeutic targeting of HMGB1 might open new therapeutic opportunities in SLE.

## Conclusions

The present study demonstrates an increase in HMGB1 levels in SLE patients, in particular in those with active renal disease. Increase in HMGB1 levels correlated to SLE disease activity index (SLEDAI) and proteinuria as well as to levels of anti-HMGB1 antibodies. Similarly anti-HMGB1 antibodies correlated to SLEDAI scores as well as to levels of anti-dsDNA antibodies. Together, we suggest that beside HMGB1, HMGB1-anti-HMGB1 immune complexes play a role in the pathogenesis of SLE.

## Abbreviations

ANA: antinuclear autoantibodies; Anti-dsDNA: anti-double stranded DNA; C3: Complement 3; C4: Complement 4; Cr: creatinine; DAMPS: damage associated molecular patterns; eGFR: estimated glomerular filtration rate; ELISA: enzyme linked immunosorbant assay; HC: healthy controls; HMGB1: High Mobility Group Box 1; RA: rheumatoid arthritis; RAGE: receptor for advanced glycation end products; SLE: Systemic Lupus Erythematosus; SLEDAI: Systemic Lupus Erythematosus Disease Activity Index; TBST: Tris buffered saline with Tween-20; TLRs: toll like receptors; TMB: 3,3',5,5'-tetramethylbenzidine dihydrochloride.

## Competing interests

The authors declare that they have no competing interests.

## Authors' contributions

DAA took part in the study design, serum HMGB1 measurement, statistical analysis, interpretation of results and manuscript preparation. JW, PCL, CGMK and MB participated in interpretation of results and manuscript preparation. JB participated in serum HMGB1 measurement, interpretation of results and manuscript preparation. CGMK participated in the interpretation of results and manuscript preparation. All authors read and approved the manuscript.
